# Energetic Assessment of the Nonexercise Activities under Free-Living Conditions

**DOI:** 10.1155/2016/8465976

**Published:** 2016-07-14

**Authors:** Shijie Sun, Qiang Tang, Haiying Quan, Qi Lu, Ming Sun, Kuan Zhang

**Affiliations:** ^1^School of Biomedical Engineering, Capital Medical University, Beijing 100069, China; ^2^Key Laboratory of Fundamental Research on Biomechanics in Clinical Application, Beijing 100069, China; ^3^Jiangsu Research Institute of Sport Science, Nanjing 210033, China; ^4^Minisun, LLC, Fresno, CA 93720, USA

## Abstract

Nonexercise activities (NAs) are common types of physical activity in daily life and critical component in energy expenditure. However, energetic assessment of NA, particularly in free-living subjects, is a technical challenge. In this study, mechanical modeling and portable device were used to evaluate five common types of NA in daily life: sit to stand, lie to sit, bowing while standing, squat, and right leg over left. A human indirect calorimeter was used to measure the activity energy expenditure of NA. Mechanical work and mechanical efficiency of NA were calculated for mechanical modeling. Thirty-two male subjects were recruited for the study (20 subjects for the development of models and 12 subjects for evaluation of models). The average (mean ± SD) mechanical work of 5 NAs was 2.31 ± 0.50, 2.88 ± 0.57, 1.75 ± 0.55, 3.96 ± 1.25, and 1.25 ± 0.51 J/kg·m, respectively. The mean mechanical efficiencies of those activities were 22.0 ± 3.3%, 26.5 ± 5.1%, 19.8 ± 3.7%, 24.0 ± 5.5%, and 26.3 ± 5.5%. The activity energy expenditure estimated by the models was not significantly different from the measurements by the calorimeter (*p* > 0.05) with accuracies of 102.2 ± 20.7%, 103.7 ± 25.8%, 105.6 ± 14.6%, 101.1 ± 28.0%, and 95.8 ± 20.7%, respectively, for those activities. These findings suggest that the mechanical models combined with a portable device can provide an alternative method for the energetic analysis of nonexercise activities under free-living condition.

## 1. Introduction

Nonexercise activities (NAs) are the physical activities other than volitional exercise, such as the activities of daily living, fidgeting, spontaneous muscle contraction, and maintaining posture when not recumbent [1]. Levine et al. pointed out that the activity energy expenditure (AEE) of nonexercise activity could result in an extra 2000 kcal of expenditure per day beyond the basal metabolic rate [2], and energy expenditure increased significantly even in fidgeting while seated (54 ± 29%) and standing (94 ± 38%) compared with metabolic rate in the supine position [3]. The AEE of nonexercise activities plays an important role in resistance to fat gain [1] and in obesity management [2]. However, energetic assessment of NA, particularly in free-living subjects, is a technical challenge.

Although a number of methods have been used to qualify NA and related AEE under free-living conditions [4–6], few of them focused on postures, transitions, and limb movements that play a very important role in daily life because of their complexity and variety. Levine et al. proposed an accelerometer method that could predict 86% of AEE for the posture and locomotion component of nonexercise activities compared with a room calorimeter but predicted only one-half of the variance in fidgeting component of nonexercise activities [7] and did not discriminate various types of fidgeting and transitions in their studies. Swartz et al. [6] established prediction models that relate hip and wrist accelerometer data to EE in filed and laboratory settings, which resulted in a minor improvement compared with models which relate only hip accelerometer data. Crouter et al. [8] examined published regression equations designed to predict energy expenditure from accelerometers over a wide range of activities and found that no single regression equation works well across a wide range of activities in light, moderate, and vigorous physical activity.

Physical activity is defined as bodily movement produced by the contraction of skeletal muscles that increases energy expenditure above the basal level and can be categorized in various ways, including type, intensity or strenuousness, and purpose [9]. Theoretically, biomechanical modeling, which has been used in human motion labs for decades, may be a fundamental approach to solve such problems. However there are some difficulties in applying the biomechanical methods under free-living conditions because there is lack of effective measurement methods of NA under free-living conditions. The traditional motion/gait analysis with force plates is relatively complicated and cumbersome, involving multiple facilities, trials and technicians, and intensive data analysis. Complexity, space, cost, and time make these analyses unacceptable for assessing daily NA of a population. Thus, mechanical models combined with portable NA measurement system are highly desired.

Mechanical work is the direct reflection of the intensity of NA from biomechanical perspective [10], and the energy expenditure can be estimated reasonably if mechanical work and mechanical efficiencies can be determined as mechanical efficiencies are defined as mechanical work/AEE. Some methods for mechanical work and mechanical efficiency calculation or prediction have been reported for walking, jogging, and swimming [11–16], but few studies were focusing on the mechanical work and mechanical efficiency of nonexercise activities under free-living conditions. In order to calculate the mechanical work of activities under free-living conditions, mathematical models in sagittal plane need to be developed with spatiotemporal parameters that can be measured under free-living condition through a portable system.

The gyroscopes of new generation are tiny, portable, accurate, and inexpensive for angular velocity measurement without affecting the signal by gravity or linear acceleration [17]; therefore, they have recently come into use in research of human movement science, such as studies of gait analysis and motion capture [18–20]. In this study, different segments angular velocities were measured by using gyroscopes. Lagrange methods were used to establish the relationship between angular parameters of different segments and the joint moments, and a joint power method was used to calculate mechanical work [10].

It is impossible to use a single mechanical model to describe daily NA, so we need to divide complicated daily NA into different subactivities and develop different models for different subactivities. In our previous study [21], we divided daily physical activities into 32 types of subactivities that could be correctly identified more than 98% by Intelligent Device for Energy Expenditure and Activity (IDEEA). In this study, five types of nonexercise activities (subactivities) were studied to test the feasibility of mechanical work calculation by using mechanical models and IDEEA system: sit to stand, lie to sit, bowing while standing, squat, and right leg over left. These NAs motions are mostly in sagittal plane and could be better described through angular kinetics and Lagrange equation. In addition, in order to explore mechanical efficiency as mechanical work/AEE for those 5 types of NA, AEE was measured while subject performed those five activities in a human indirect calorimeter.

## 2. Methods

### 2.1. The Development of Mechanical Models

**Figure 1:**
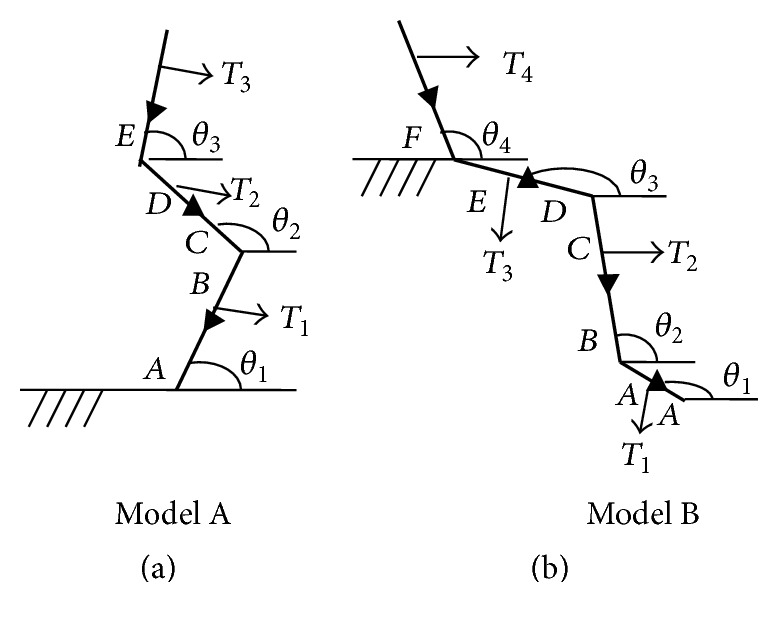
Link segment Models A and B showing limb angles, torques, and limb length variables (small triangles indicate centers of gravity).

Two mechanical models of NA were developed in this study: Model A for sit to stand (STS), bowing while standing (BOW), and squat (SQ) and Model B for lie to sit (LTS) and right leg over left (RLOL) (Figures 1(a) and 1(b)). Model A is a three-segment model with the shank, thigh, and trunk. The feet (ankles) are assumed to be fixed on the ground considered as the ordinate origin in the global reference system. In Model A, there are three angular variables *θ*_1_, *θ*_2_, and *θ*_3_ that represent the angles of shank, thigh, and trunk against ground corresponding to variables *T*_1_, *T*_2_, and *T*_3_ that represent the joint torques of ankle, knee, and hip joints.

Model B is a four-segment model with the foot, leg, thigh, and trunk. The hip is assumed to be fixed onto a chair and the bed is considered as the ordinate origin in the global reference system. The upper body and lower body are considered as separated parts when calculating and the hip joint torques as *T*_3_ and *T*_4_. In Model B, there are four angular variables *θ*_1_, *θ*_2_, *θ*_3_, and *θ*_4_ that represent the angles of foot, shank, thigh, and trunk against the ground corresponding to four joint torques *T*_1_, *T*_2_, *T*_3_, and *T*_4_.

In both Models A and B, torques can be derived using Lagrange's equations with angular variables [10]:

(1) $$\begin{array}{c} \frac{\partial \left(\partial L/\partial {\dot{q}}_{i}\right)}{\partial t}-\frac{\partial L}{\partial {\dot{q}}_{i}}=Q_{i},\;\left(i=1,\ldots ,n\right). \end{array}$$

The equations for Model A can be described as follows with second derivatives on the left:

(2) $$\begin{array}{c} \left[\begin{array}{ccc} a_{11} & a_{12} & a_{13}\\ a_{21} & a_{22} & a_{23}\\ a_{31} & a_{32} & a_{33} \end{array}\right]\left[\begin{array}{c} {\ddot{\theta }}_{1}\\ {\ddot{\theta }}_{2}\\ {\ddot{\theta }}_{3} \end{array}\right]=\left[\begin{array}{c} b_{1}\\ b_{2}\\ b_{3} \end{array}\right] \end{array}$$

or

(3) $$\begin{array}{c} \left[\begin{array}{c} a_{ij} \end{array}\right]{\ddot{\theta }}_{i}=b_{i}. \end{array}$$

The term *a*_*ij*_ consists of anthropometric constants and the segment angles:

(4) $$\begin{array}{c} a_{ij}=c_{ij}\cos\left(\;\theta_{i}-\theta_{j}\;\right), \end{array}$$

*b*_*i*_ consists of joint torques, and *c*_*ij*_ is the square of angular velocities. *m*_*s*_, *m*_*t*_, and *m*_*h*_ are the mass of shank, thigh, and HAT (head, arms, and trunk), *I*_*s*_, *I*_*t*_, and *I*_*h*_ are the moment of inertia about the center of gravity of the shank, thigh, and HAT, and *g* is the gravitational constant. Consider

(5) c11msA2+mtA+B2+mhA+B2+Isc12c21=mtA+BC+mhA+BC+Dc13c31=mhA+BEc22mtC2+mhC+D2+Itc23c32=mhC+DEc33mhE2+Ihb1T1−T2−msgA+mt+mhgA+Bcos⁡θ1−∑i=13c1isin⁡θ1−θiθ˙i2b2T2−T3−mtgC+mhgC+Dcos⁡θ2−∑i=13c2isin⁡θ2−θiθ˙i2b3T3−mhgEcos⁡θ3−∑i=13c3isin⁡θ3−θiθ˙i2.

The same definitions in Model A apply to Model B with similar equation

(6) $$\begin{array}{c} \left[\begin{array}{cccc} a_{11} & a_{12} & a_{13} & 0\\ a_{21} & a_{22} & a_{23} & 0\\ a_{31} & a_{32} & a_{33} & 0\\ 0 & 0 & 0 & a_{44} \end{array}\right]\left[\begin{array}{c} {\ddot{\theta }}_{1}\\ {\ddot{\theta }}_{2}\\ {\ddot{\theta }}_{3}\\ {\ddot{\theta }}_{4} \end{array}\right]=\left[\begin{array}{c} b_{1}\\ b_{2}\\ b_{3}\\ b_{4} \end{array}\right] \end{array}$$

or

(7) $$\begin{array}{c} \left[\begin{array}{c} a_{ij} \end{array}\right]{\ddot{\theta }}_{i}=b_{i}. \end{array}$$

The details of terms *a*_*ij*_ and *b*_*i*_ are as formula (9). *m*_*f*_, *m*_*s*_, *m*_*t*_, and *m*_*h*_ are the mass of foot, shank, thigh, and HAT and *I*_*f*_, *I*_*s*_, *I*_*t*_, and *I*_*h*_ are the moment of inertia about the CG of the foot, shank, thigh, and HAT. Consider

(8) aijcijcos⁡θi−θj

(9) c11mfA2+Ifc12c21=mfAB+Cc13c31=mfAD+Ec22msC2+mfB+C2+Isc23c32=msD+EC+mfD+EB+Cc33mtE2+ms+mfD+E2+Itc44mhF2+Ihb1−T1+mfgAcos⁡θ1−∑i=13c1isin⁡θ1−θiθ˙i2b2T1−T2−msgC+mhgB+Ccos⁡θ2−∑i=13c2isin⁡θ2−θiθ˙i2b3T2−T3+mtgE+ms+mfgD+Ecos⁡θ3−∑i=13c3isin⁡θ3−θiθ˙i2b4T4−mhgFcos⁡θ4.

From the equations above, the joint torques can be calculated according to the angular variables and anthropometric data. Therefore the mechanical work of designed five types of NAs can be calculated with joint power and work method:

(10) Pi,t=Ti,tθ˙i,tWt=∫t1t2∑i=1nPi,tdt,

where *P*_*i*,*t*_, *T*_*i*,*t*_, and ${\dot{\theta }}_{i,t}$ are the joint power, torque, and angular velocity of the *i*th joint at the time *t*, respectively, and *W*_*t*_ represents the total work of all the segments from time *t*_1_ to time *t*_2_. *n* is the number of joint moments. For Model B, *n* = 4 because hip joint moment consists of the moment caused by trunk and the moment caused by thigh. The total mechanical work (MW) is calculated by summing the power curves of all joints, in a way which allows energy transfer among joints and only the positive part of total MW was used to derive mechanical efficiency (ME) in this study.

### 2.2. Angular Parameters Measurement

A customized Intelligent Device for Energy Expenditure and Activity (IDEEA) with eight uniaxial gyroscopes (64 Hz, Minisun LLC, CA, USA) was used to measure angular velocities of foot, shank, thigh, and HAT.

Foot gyroscope was placed about 2 cm below ankle joint and shank gyroscope was placed about 3 cm above ankle joint to minimize the interference by muscle fasciculation. Thigh gyroscope was placed in the middle of the thigh and HAT gyroscope was placed in the middle of right side of trunk. Gyroscope data was filtered with a 4th-order low-pass Butterworth filter with a 4 Hz cutoff frequency.

The signal from gyroscopes represents the angular velocity as ${\dot{\theta }}_{i}$ during each type of NA. The angles (*θ*_*i*_) are calculated by integration of gyroscope signals and a moving average filter was used to correct the drift of angles. At the beginning of each trial, subjects were asked to maintain posture for 5 seconds to measure the baseline (sitting still with trunk upright for STS and RLOL trial; lying supine for LTS trial; standing still in an upright position for SQ and BOW trial) [17]. ${\ddot{\theta }}_{i}$ was calculated by derivation of gyroscope signals.

**Figure 2:**
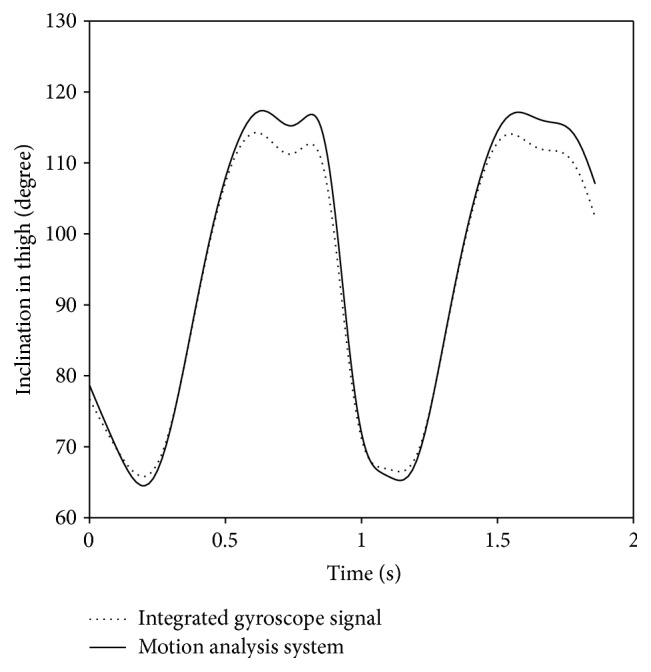
Evaluation of the performance of gyroscopes by comparing integrated inclination data on the thigh with motion analysis system.

A motion analysis system (Motion Analysis, CA, USA) was used to evaluate the measurement of gyroscopes during walking of one randomly selected subject. Thigh inclination data measured by a gyroscope and thigh inclination data from the motion analysis system are shown in Figure 2. The correlation coefficient of two curves is 0.998, and the root mean square error (RMSE) is 3.16 degrees.

### 2.3. Energy Expenditure Measurement

Energy expenditure (EE) was measured in a human indirect calorimeter (Minisun, CA, USA) at Research Institute of Sport Science, Jiangsu, China. The calorimeter is 2.7 × 3.0 × 2.4 m room. The temperature was controlled at 25.0 ± 1.0°C. The subject's O_2_ consumption and CO_2_ production were determined from the measured flow rate and the differences in O_2_ and CO_2_ concentrations between entering and exiting air. Metabolic rate (MR) is provided on minute-by-minute basis. Repeated alcohol burn experiments yielded carbon dioxide and oxygen recoveries of ≈98% from the room calorimeter, confirming errors of energy expenditure measurements within 2%.

### 2.4. Inertial Parameter Selection for Mechanical Work Calculation

Inertial parameters were calculated according to the height and weight of subjects with regression equation reported in Chinese inertial parameter of adult human [22]. For STS, LTS, BOW, and SQ activities, inertial parameters from right and left thigh and shank were applied while only inertial parameters from right shank and foot were applied for RLOL activity.

### 2.5. Subjects

Thirty-two healthy male subjects (23 ± 4 yr; 72.2 ± 12.7 kg; body mass index 22.9 ± 3.1 kg/m^2^) were recruited for the study. They were presented with detailed description of the experimental protocol and were asked to sign consent forms approved by the Ethics Committees of Research Institute of Sport Science, Jiangsu, China, and Capital Medical University, Beijing, China. Subjects had no neurological or musculoskeletal impediments.

### 2.6. Experimental Protocol

Fasted and rested subjects were admitted into the indirect room calorimeter after being fitted with the IDEEA with gyroscopes. Subjects were required to follow the protocol in Table 1.

Before the NA trials, baseline metabolic values were quantified while subjects sat quietly in a chair for 20 min. Because ME may be influenced by different frequencies of motions and some studies have shown that the highest ME occurred at preferred motion frequency [14], when subjects performed designed NAs, the motion was repeated at self-controlled and comfortable frequencies. There were 10 min break between NAs to allow metabolic values to return to the baseline.

### 2.7. Statistics

All results are presented as mean ± SD. For each randomly selected subject, the EE and MW for each minute of experimental protocol were calculated.

The activity energy expenditure (AEE) of each NA was calculated as AEE = total EE − resting EE. The ME of each NA was calculated as ME = MW/AEE. For each set of randomly selected 20 subjects, mean MW, AEE, and ME of each NA were calculated. MW and AEE were described by unit mass and unit height. Linear regression analysis was used to determine if ME is affected by height or weight.

The data of the remaining 12 subjects, who were not used in modeling, were used to evaluate the mathematical models. AEE estimated from MW and ME were compared with AEE measured by the calorimeter. The accuracy was used to compare the estimated AEE by the models with AEE from the calorimeter: accuracy = (AEE from models)/(AEE from calorimeter). A post hoc paired *t*-test (with the Bonferroni correction) was used to detect the difference between the estimated and measured EE and the intraclass correlation coefficient was calculated (corrected for the fixed bias that exists between the two methods). The statistics program SPSS 19.0 (SPSS Inc., Chicago, USA) was used. Statistics significance was defined as *p* < 0.05.

## 3. Results

The 20 randomly selected subjects for the development of models are with average age of 23.1 ± 4.6 (mean ± SD) years, weight 75.4 ± 13.9 kg, height 1.79 ± 0.07 m, and body mass index (BMI) 23.5 ± 3.5 kg/m^2^. The mean MW, AEE, and ME of those 20 subjects are shown in Table 2. Previous study has suggested that AEE is related to both height and weight [23]; therefore in Table 2 MW and AEE are described by unit mass and unit height, and the relationship between mechanical efficiencies and height or weight is explored. The results show that there are no significant correlations between mechanical efficiencies of 5 NAs and body weights (*r* ^2^ = 0.051, 0.048, 0.000, 0.004, and 0.005; *p* = 0.785, 0.353, 0.951, 0.314, and 0.320, resp.) or height (*r* ^2^ = 0.139, 0.062, 0.006, 0.105, and 0.033; *p* = 0.059, 0.150, 0.749, 0.163, and 0.443, resp.).

**Figure 3:**
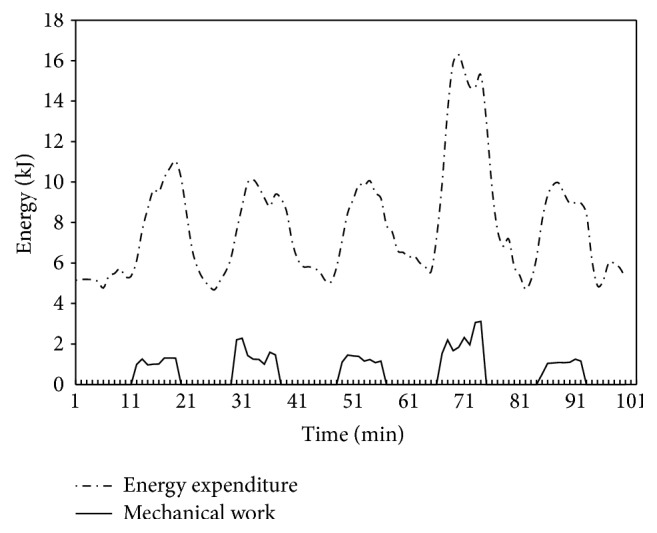
Energy expenditure and mechanical work curve of one subject in experimental protocol.

**Figure 4:**
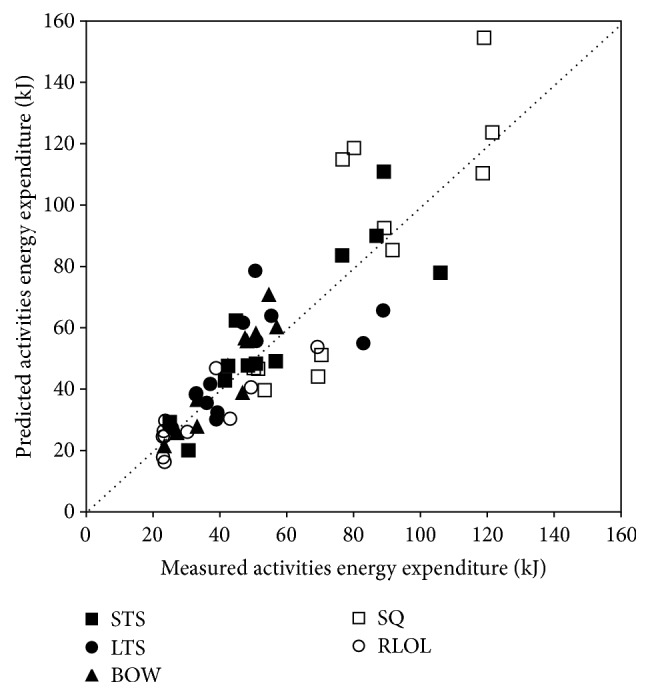
Predicted AEE versus measured AEE. The symbols represent different types of PA in 12 subjects. The dotted line is the line of identity.

The MW and AEE curves from one subject with 100-minute performance of 5 sets of NAs are shown in Figure 3 (*r* = 0.793). For the remaining 12 subjects, the measured average AEE for each NA were 58.27 ± 25.43, 48.83 ± 19.30, 40.89 ± 11.73, 82.66 ± 26.12, and 33.64 ± 14.40 kJ. The estimated average AEE were 59.11 ± 26.56, 48.82 ± 16.68, 43.95 ± 16.26, 85.67 ± 39.15, and 31.25 ± 11.41 kJ, respectively. The post hoc paired *t*-test shows that there is no significant difference between estimated AEE and measured AEE (*p* = 0.827, 0.997, 0.141, 0.694, and 0.304), and the ICC is 0.939, 0.793, 0.935, 0.882, and 0.903, respectively. The accuracies of estimated AEE for those 5 NAs are 102.2 ± 20.7%, 103.7 ± 25.8%, 105.6 ± 14.6%, 101.1 ± 28.0%, and 95.8 ± 20.7%, respectively.  Figure 4 shows the comparison betweenestimated AEE and measured AEE. A highly significant correlation was found (*r* = 0.877, 0.644, 0.937, 0.841, and 0.847; *p* < 0.05) between estimated AEE and measured AEE. For the two-tailed test with significant criterion *p* < 0.05, the statistics power for the AEE modeling in the Chamber tests (during 100 min protocol; *r* = 0.856; sample size *N* = 20) is 0.98 and for the AEE evaluation of models (during 100 min protocol; *r* = 0.922; sample size *N* = 12) is 0.95. The hypothesis that the regression slopes in Figure 4 are different from 1.0 has been tested and rejected (*p* = 0.721, 0.629, 0.211, 0.897, and 0.496, resp.).

## 4. Discussion

In this study, MW and ME for 5 NAs have been calculated based on the biomechanical models. The result shows that an alternative method can be provided for the energetic analysis of nonexercise activities under free-living condition. Nonexercise activities, including postural transitions and fidgeting, are very important components of daily physical activities. The AEE of daily nonexercise activities is highly variable not only among people with sedentary occupations and nonsedentary occupations but even among individuals who are sedentary [24]. Few literatures focused on the MW and ME of nonexercise activities in previous studies and results of MW and ME of 5 NAs from this study could provide new perspective for human activities exploration.

Measuring the level of these NAs is a complex task, especially under free-living conditions. In our previous study, we could identify various nonexercise activities, but we found it difficult to estimate intensities of transition movements and fidgeting-like NAs [25]. In this study, this problem was analyzed from biomechanical perspective, through mechanical work and efficiency calculations. Relationship between AEE and MW curves shown in Figure 3 indicates that a possible method for estimating AEE from MW could be calculated from models.

It is difficult for a room calorimeter to measure the energy expenditure of a single cycle of NA because of body storage of oxygen and carbon dioxide [26]; therefore, we had to measure each NA for a longer period. After being standardized for number of cycles of each NA, weight, and height, the MW and the AEE of each NA in 20 subjects are shown in Table 2; thus a simple estimating method for MW and AEE of nonexercise activities could be provided according to the movement frequency, body weight, and height. Júdice et al. [27] measured EE of STS in a calorimeter (1.49 ± 0.25 kcal/min) in 2015. According to the resting energy expenditure (1.14 ± 0.18 kcal/min) and the movement frequency (one cycle per minute) referred to in their work, the AEE of one STS cycle was about 1463.7 J (after unit conversion), which was similar to the result of this study (1453 ± 524 J).

Rising from a chair has been regarded as the most mechanically demanding task undertaken during daily activities [28], and STS movement has been reported to be repeated about 60 times per day for healthy adults [29]. In the previous studies, mechanical analysis of STS mainly focused on the kinetic and kinesiology parameter and few literatures reported mechanical work calculation or energy expenditure measurement. MW and AEE are important aspects for evaluating physical activity, so MW and AEE were analyzed to assess STS in this study.

The mean efficiencies of 5 NAs from this study derived from first 20 subjects are in the physiological range between 10% and 30% [30]. The results from this study also show that the efficiencies of each type of NA were consistent for different weight and height, which makes it possible to assume a constant efficiency for each type of NA with comfortable frequency (22.0% for STS; 26.5% for LTS; 19.8% for BOW; 24.0% for SQ; 26.3% for RLOL). To test the possibility, AEE of STS, LTS, BOW, SQ, and RLOL cycles from other 12 subjects were estimated based on ME derived from first 20 subjects, respectively. Result shows significant correlations between estimated and measured AEE. The average accuracies of different NAs are close to 100%; however, the SD of the accuracies is large (±20.7%, ±25.8%, ±14.6%, ±28.0%, and ±20.7%); the large SD of accuracies is attributable to the large variances of MEs, which could have individual differences up to 16%. The similar individual differences of ME (about 15%) were found in other studies [14]. In the further study, larger sample size and individual calibration methods could be employed to reduce the SD and improve the accuracy and external validity.

There were some limitations in this study. First, only five types of NA were examined in this study and, in the future, more types of NAs should be considered for the study of daily NA. Second, only 32 young and healthy Asian male subjects were recruited in this preliminary study for methodology exploration. In the future, larger sample size is needed and differences in gender, age, health, ethnicity, and other population characteristics need to be considered.

## 5. Conclusion

The mean efficiencies of STS, LTS, BOW, SQ, and RLOL were 22.0 ± 3.3%, 26.5 ± 5.1%, 19.8 ± 3.7%, 24.0 ± 5.5%, and 26.3 ± 5.5%, respectively, and could be applied to estimate AEE of corresponding activities at preferred motion frequencies. By use of biomechanical models and portable measurement device of segment angular velocity, MW and AEE of five nonexercise activities could be calculated and estimated, which may provide an alternative method for studying daily NA for free-living subjects.

## Figures and Tables

**Table 1:** Sequence and duration of PAs in room calorimeter.

Duration	Type of PA
20 min	Sat quietly
8 min	STS
10 min	Sat quietly
8 min	LTS
10 min	Sat quietly
8 min	BOW
10 min	Sat quietly
8 min	SQ
10 min	Sat quietly
8 min	RLOL
10 min	Sat quietly

**Table 2:** MW, AEE, and ME of STS, LTS, BOW, SQ, and RLOL for 20 subjects.

Type of PA	Total MW (J/kg·m)	AEE (J/kg·m)	ME (%)
STS	2.31 ± 0.50	10.6 ± 2.3	22.0 ± 3.3%
LTS	2.88 ± 0.57	11.2 ± 3.0	26.5 ± 5.1%
BOW	1.75 ± 0.55	9.1 ± 3.5	19.8 ± 3.7%
SQ	3.96 ± 1.25	17.2 ± 6.8	24.0 ± 5.5%
RLOL	1.25 ± 0.51	4.8 ± 1.9	26.3 ± 5.5%
